# Soft Finger Modelling and Co-Simulation Control towards Assistive Exoskeleton Hand Glove

**DOI:** 10.3390/mi12020181

**Published:** 2021-02-11

**Authors:** Mohammed N. El-Agroudy, Mohammed I. Awad, Shady A. Maged

**Affiliations:** 1 Mechatronics Engineering Department, Faculty of Engineering, Ain Shams University, Cairo 11566, Egypt; mohammed.ibrahim.nagy@gmail.com (M.N.E.-A.); mohammed.awad@eng.asu.edu.eg (M.I.A.); 2 Mechatronics Engineering Department, Faculty of Engineering, Galala University, Cairo 11566, Egypt

**Keywords:** soft robotics, rehabilitation, exoskeleton, hand glove, finite element modeling, software-in-the-loop, design of experiment

## Abstract

The soft pneumatic actuators of an assistive exoskeleton hand glove are here designed. The design of the actuators focuses on allowing the actuator to perform the required bending and to restrict elongation or twisting of the actuator. The actuator is then modeled using ABAQUS/CAE, a finite element modeling software, and the open loop response of the model is obtained. The parameters of the actuator are then optimized to reach the optimal parameters corresponding to the best performance. Design of experiment (DOE) techniques are then approached to study the robustness of the system. Software-in-the-loop (SiL) is then approached to control the model variables via a proportional-integral-derivative (PID) control generated by FORTRAN code. The link between the two programs is to be achieved by the user subroutine that is written, where the subroutine receives values from ABAQUS/CAE, performs calculations, and passes values back to the software. The controller’s parameters are tuned and then the closed loop response of the model is obtained by setting the desired bending angle and running the model. Furthermore, a concentrated force at the tip of the actuator is added to observe the actuator’s response to external disturbance.

## 1. Introduction

Our hands play a vital role in our daily routine; whether for a 10-year-old or even a 60-year-old, they are irreplaceable. Unfortunately, though, factors such as aging, accidents, strokes, and other factors can cause losses in some of the functionalities, which require the patient to go through rehabilitation. These physical therapy sessions require the patient to go through several stages of exercises to gradually increase the strength of the muscles and to regain the ability to perform the common daily functions of the hand. These sessions are commonly monitored by a physical therapist, who designs a recovery plan for the patient, guides the patient during performing the tasks and assesses how the patient responds. This process of course takes time and money, as it can take up to 6 months. Thus, an at-home rehabilitation device is needed.

An exoskeleton hand glove can solve these problems. The patient can use it at home without needing to go to the hospital or to the physical therapist. It can record the performance of the patient and their progress and sends the data to the doctor to assess and monitor the performance of the patient. Such a glove would save money and time, and would allow swift communication between the doctor and the patient.

Upon designing the glove, we chose to actuate it with soft pneumatic actuators (SPAs). This type is chosen as it can comply with the fingers easily, and thus distributes the force over the finger. Additionally, the finger’s different motions are studied. Human fingers can perform flexion, extension, adduction, abduction, opposition and reposition [1]. Accordingly, SPAs can be correctly designed.

The SPA is supplied with air pressure to perform the required bending motion. Upon increasing the inlet pressure, the bending of the SPA increases. Such pressure must be controlled by a sophisticated controller that controls the amount of air pressure supplied to the actuator based on the current actuator angle.

In the last decade, many researchers have started to model and fabricate SPAs to use in the rehabilitation glove, such as in Refs. [2,3,4,5,6,7,8,9,10]. Panagiotis Polygerinos et al. modeled, fabricated and controlled soft pneumatic actuators that are constructed from a combination of elastomeric materials and inextensible materials [2], and discussed the control of the SPA using electromyography (EMG) sensors for sensing user intent and measuring muscle response, in Ref. [3]. This study shows how to create soft actuators and how the outputs change as a function of input pressure. Hong Kai Yap et al. presented the performance and characteristics of an SPA that is used in a rehabilitation glove, with different materials and radii of curvature [4], and they designed a soft wearable robotic device for rehabilitation and assistance using SPAs with variable stiffness in Ref. [5]. In Ref. [6], Philip Moseley et al. modeled, designed, and developed a soft pneumatic actuator with the finite element method. In Ref. [7], Atta Oveisi et al. performed a finite-element (FE)-based software-in-the-loop control, wherein the FE model was controlled by MATLAB. Zheng Wang et al. developed a quasi-static analytical model based on the bending moments generated by the applied internal pressure, and created an FE model [8]. In Ref. [9], Khaled Elgeneidy et al. presented a data-driven modeling approach for predicting and controlling the bending angle response of an SPA. Y. Jiang et al. proposed a novel fabrication method for an SPA that combines the advantages of the lost-wax technique and inverse-flow-injection processes [10]. Most researchers use SPA to actuate the glove through an air compressor, whereby the actuator performs flexion and force is applied on the finger, forcing the finger to bend.

In this manuscript, an FE model of an SPA is constructed. The dimensions of the actuator are then optimized to select the optimum parameters. The individual and interactive effects of the design parameters are then studied through a design of experiment to verify the robustness of the model. PID control is then applied on the FE through a FORTRAN code. Such control of the FE model can show us the dynamic response of the actuator to the controlled input.

This paper is organized as follows: Section 2 presents the materials and methods, including the design, the FE model, the optimization of the SPA, and the design of experiment and software-in-the-loop by linking the FE model with the control. The results will be shown in Section 3. Finally, the discussion and concluding remarks will be presented in Section 4.

## 2. Materials and Method

**Figure 1:**
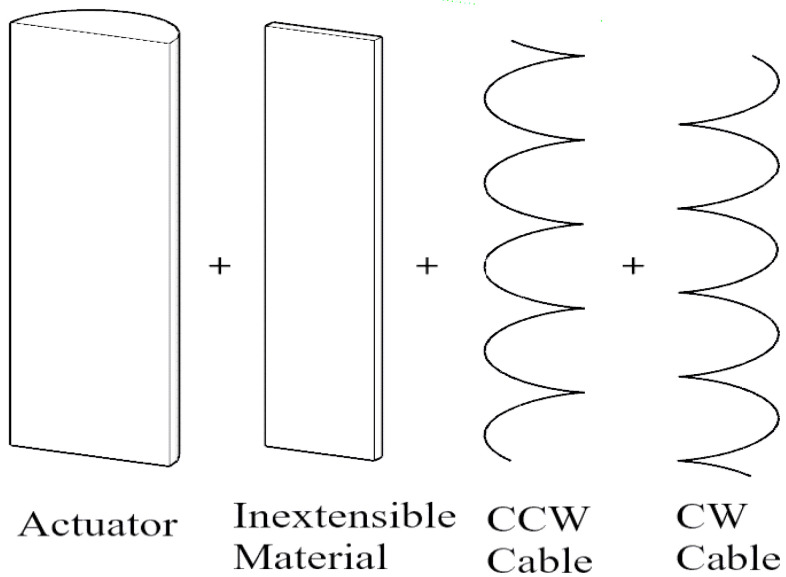
Bending design of soft pneumatic actuator.

**Figure 2:**
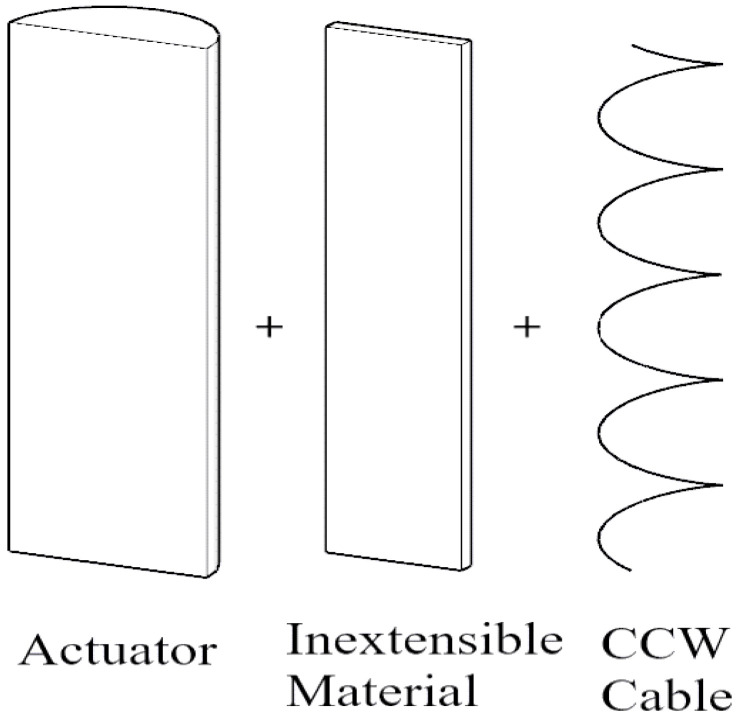
Twisting design of soft pneumatic actuator.

**Figure 3:**
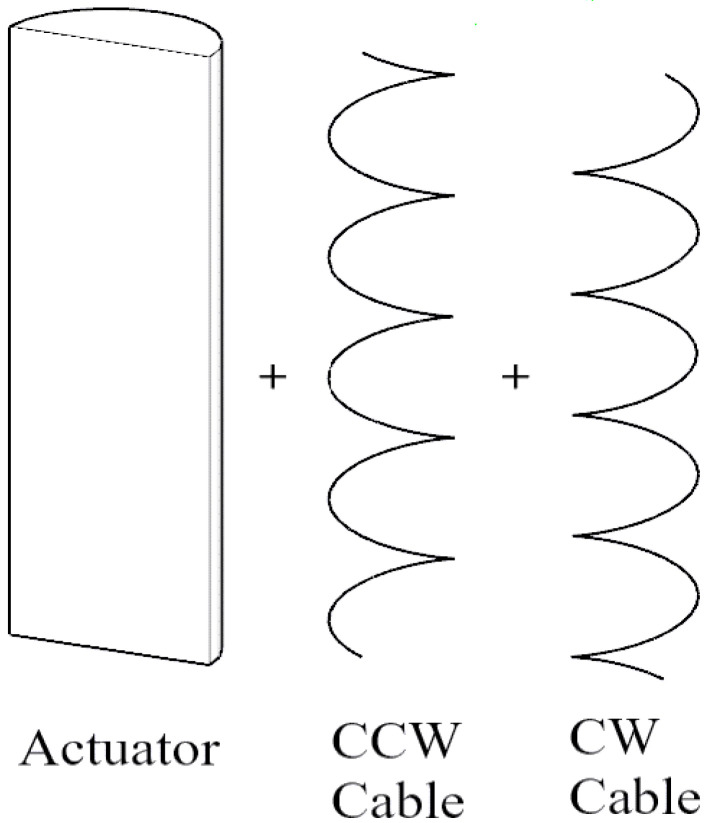
Extension design of soft pneumatic actuator.

The design consists of soft actuators made of silicone. The soft actuators when pressurized tend to move in any direction. To make the actuator go in a specific direction, degrees of freedom must be restricted so that the actuator moves in the other non-restricted directions. For an actuator to bend, the actuator must be prevented from expanding, extending, and twisting in any direction so that it can only bend. This can be done by wrapping Kevlar cable, in both clockwise and anti-clockwise directions, around the actuator to prevent twisting, and by attaching fiberglass to the actuator to prevent extension, as in Figure 1. For an actuator to twist in one direction, the Kevlar cable is only wrapped in one direction, opposite to the desired direction of twisting, and also fiberglass is attached to prevent extension, as in Figure 2. For an actuator to extend, as in Figure 3, Kevlar cable is wrapped in both clockwise and counterclockwise directions to prevent twisting [2,3].

**Figure 4:**
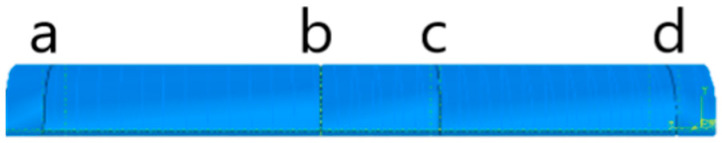
Thumb segmentation.

**Figure 5:**
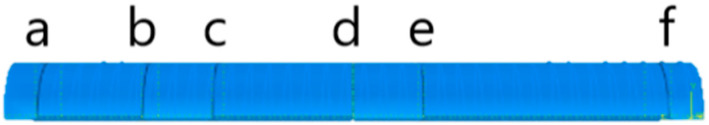
Index segmentation.

To mimic the fingers’ motion, a segmented actuator design is approached. The segmented actuator is designed so that the actuator can extend at the finger’s joints, and bend at the remaining parts of the finger. This novel design allows the actuator to comply with the fingers’ anatomy. The thumb is divided into 3 segments; the first segment is for twisting and bending, to cover the part of the thumb that twists. The second segment is for elongation, to cover the joint of the thumb so that it extends, compensating for the actuator’s length so that it stays compliant with the thumb. The third segment is for bending [11], to cover the rest of the finger, as shown in Figure 4. The index is divided into five segments. The two segments above the joints are for extending and bending, to cover the joints and extend when the joints are bent so that the actuator stays compliant with the finger. The other three segments are for bending, covering the rest of the finger, as shown in Figure 5. The middle, ring and little finger are designed similarly to the index, but with different lengths of the segments.

**Figure 6:**
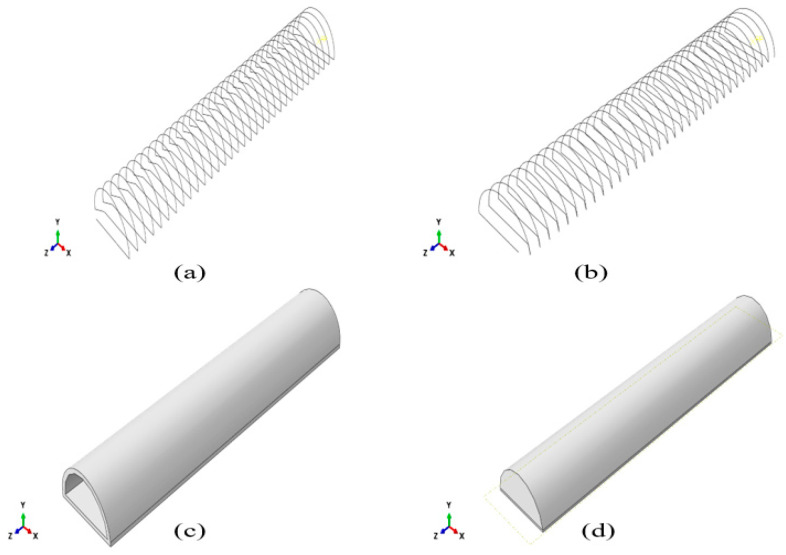
FE model of each of the actuator’s parts. (**a**) Counterclockwise cable. (**b**) Clockwise cables. (**c**) Outer layer. (**d**) Tube.

**Figure 7:**
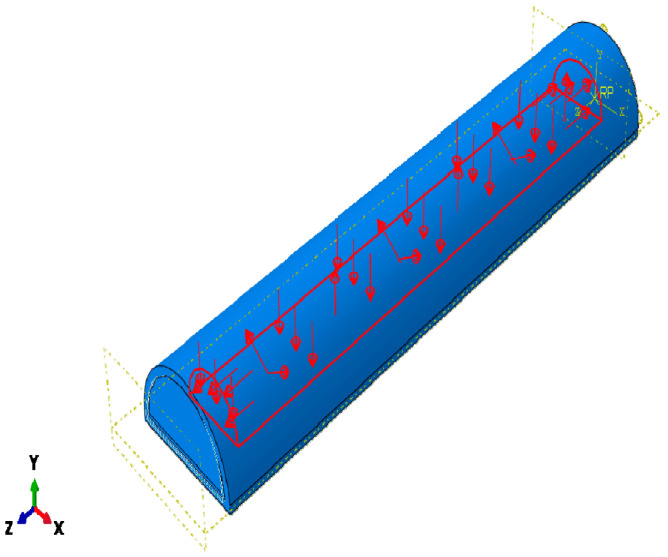
Load applied to the inner surface of the SPA.

The SPA was modeled on ABAQUS/CAE (2019, Dassault Systèmes^®^, Vélizy-Villacoublay, France) [12,13,14]. The model is drawn as 4 parts: tube, outer layer, clockwise cable, and anti-clockwise cables, as shown in Figure 6. The tube has a semi-circular cross-section [15]. It is also segmented so that 0.5 mm from the bottom is non-extendable, using a fiber glass material to prevent the actuator from elongating. Additionally, it is extruded 5 mm from each side so that it is solid from both sides and has a cavity where the pressure is applied. The Kevlar cables are added by a python script, in both clockwise and anticlockwise directions, to prevent the actuator from twisting in either direction. Materials are assigned to each part by modeling each material property. The tube material is selected to be Dragon-Skin 20 [16]. It is modeled as a Neo-Hookean hyperplastic material. The outer layer material is selected to be Dragon-Skin 10 [17], which is also modeled as a Neo-Hookean hyperplastic material. Boundary conditions are applied on the actuator so that one of its faces is fixed. Pressure is applied to the inner surface of the SPA, as shown in Figure 7.

**Figure 8:**
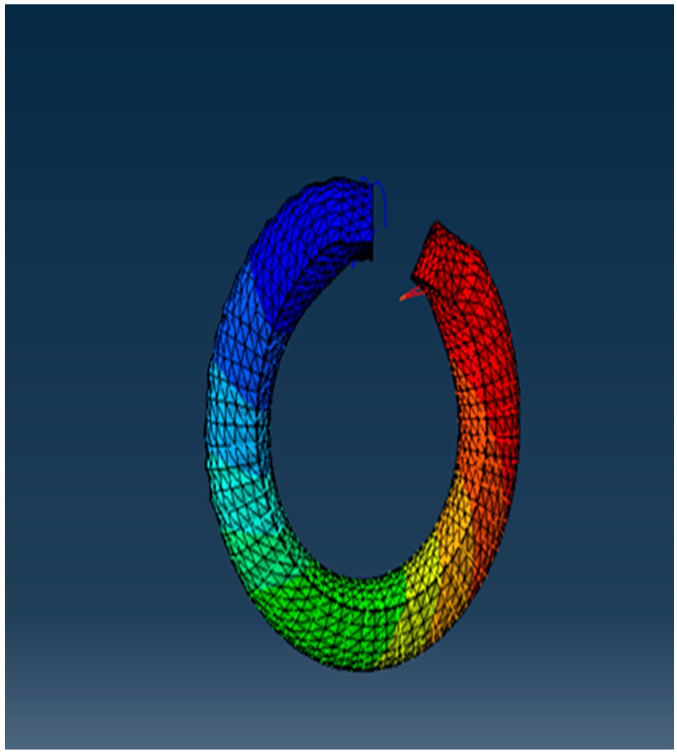
Open loop simulation of the SPA.

The model is first modeled with an equally spaced amplitude step to verify the model’s behavior. A hybrid element mesh type is used since the material is a hyper elastic material. The job is submitted and the results are extracted. The actuator bends as expected, as shown in Figure 8. The open loop simulation results are presented in Section 3.1.

**Figure 9:**
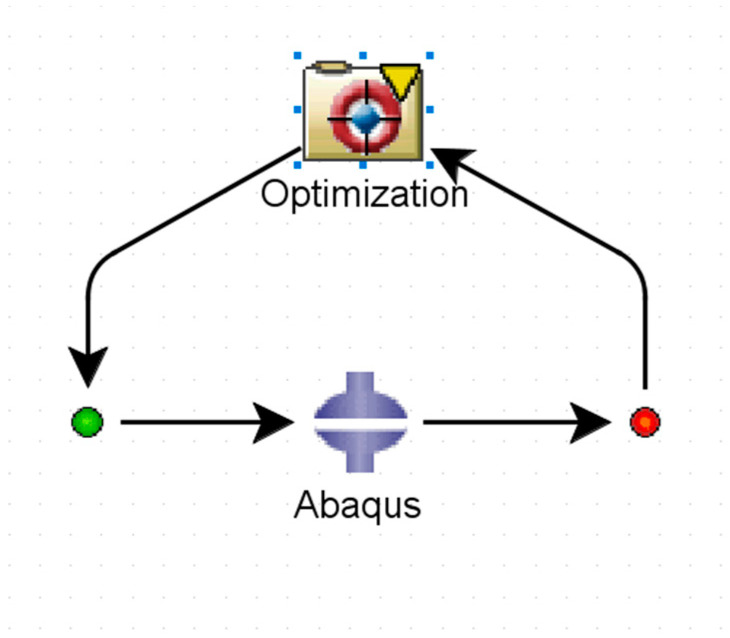
Optimization process loop.

Data for both maximum Von Mises stress and maximum displacement can be extracted. The design of the glove can be optimized to find the optimal values that give the best actuator response. ISight^®^ (2019, Dassault Systèmes^®^, Vélizy-Villacoublay, France ) is used for solving the optimization problem and ABAQUS/CAE is added in ISight^®^ so that ISight^®^ can work on optimizing the model. A multi-objective exploratory optimization technique called the neighborhood cultivation genetic algorithm (NCGA) is selected to work on the model [18]. The optimization task takes the output values (maximum stress, maximum displacement, etc.) from the ABAQUS/CAE component that contains the bending design of the SPA, performs the optimization iteration, and returns the parameters being optimized (dimensions, materials, etc.). This loop is shown in Figure 9.

**Figure 10:**
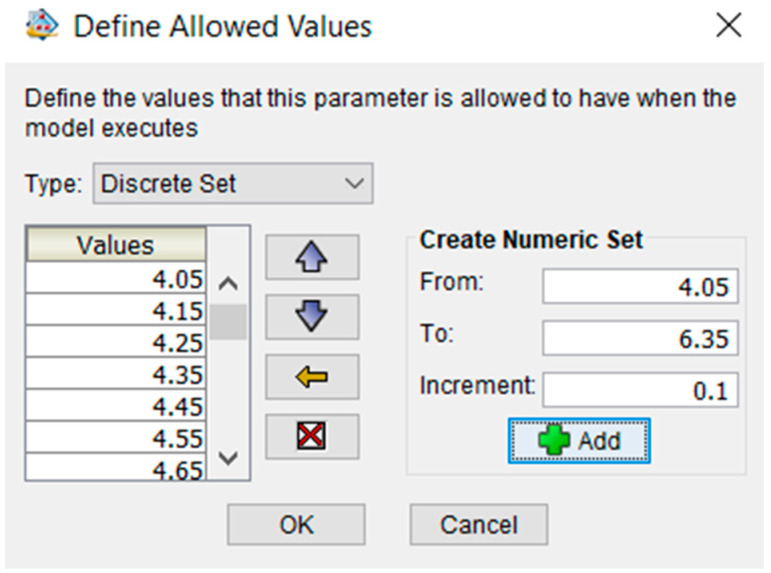
Defining allowed values for the SPA’s inner radius.

**Figure 11:**
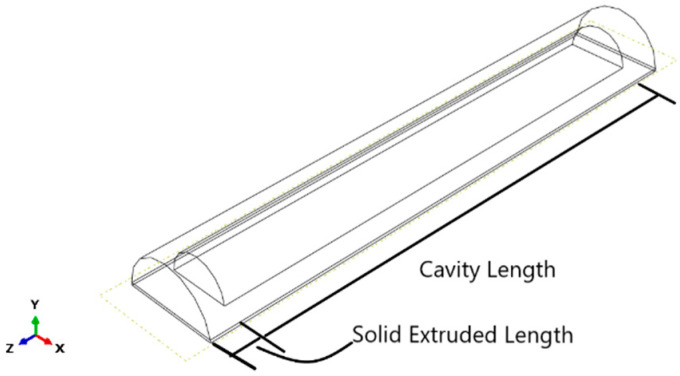
The relation between solid extruded length and cavity length.

First, a dimensional optimization problem is solved, where the input parameters are selected to be the inner radius and the solid extruded part at the tube’s tip, which reflect the cavity length of the tube. The inner radius is given values from 4.05 mm to 6.35 mm with 0.1 mm increments, as shown in Figure 10. The solid extruded length also allowed for values belonging to a discrete set but from 3 mm to 5 mm with 0.1 mm increments, which reflects the cavity length changing from 85 mm to 87 mm with 0.1 mm increments, as shown in Figure 11.

**Figure 12:**
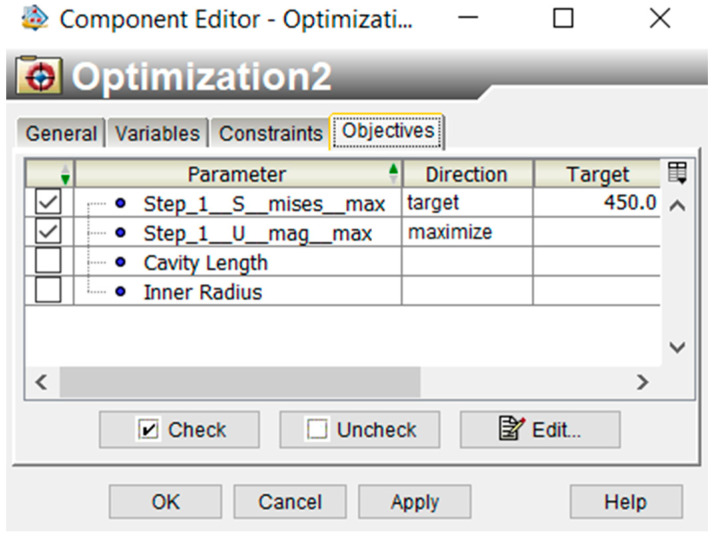
Objective selection.

The objectives are then chosen. The first objective is to maximize the displacement of the actuator. The second objective is for the maximum stress point to target a value of 450 MPa, as shown in Figure 12.

Another model is also solved with the same objective function, but with input parameters set as the inner radius and outer layer thickness. The inner radius has the same given values and the outer layer thickness ranges from 0.5 mm to 5 mm, with 0.5 mm increments. Furthermore, the material optimization of the actuator is addressed by selecting the C10 coefficients of inner and outer layer materials as input parameters. C10 is a coefficient that depends on the material properties, such as young modulus. The allowed values are 0.031648 for Dragon-Skin 20, 0.0425 for Dragon-Skin 10, 0.03 for EcoFlex-50 and 0.12 for Elastosil. The objective function is the same as in the dimensional optimization model. The optimization results are presented in Section 3.2.

After reaching the optimal parameters for the system, these parameters are investigated to verify the robustness of the system and to determine the individual and interactive effects of the design parameters.

**Figure 13:**
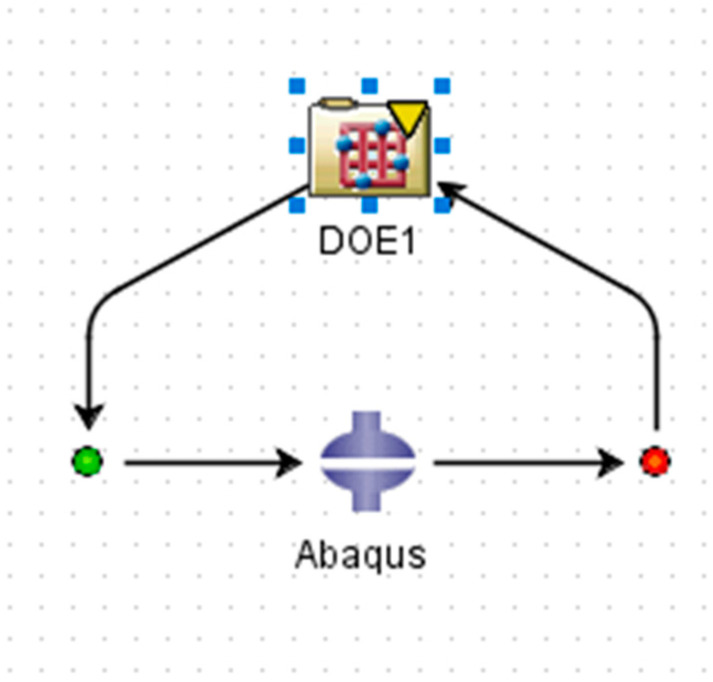
DOE loop.

**Figure 14:**
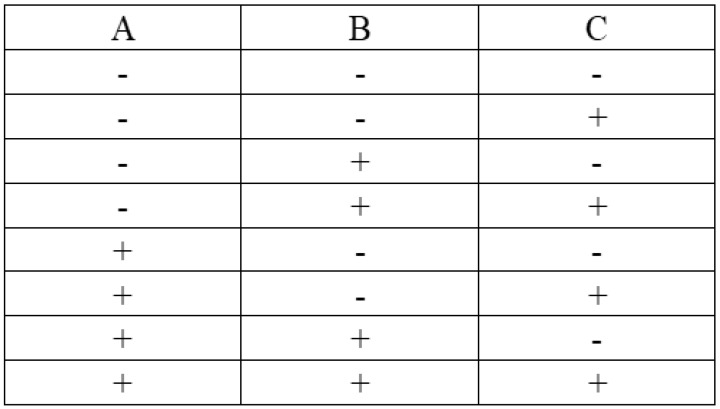
Full factorial two-level three-factor design matrix.

**Figure 15:**
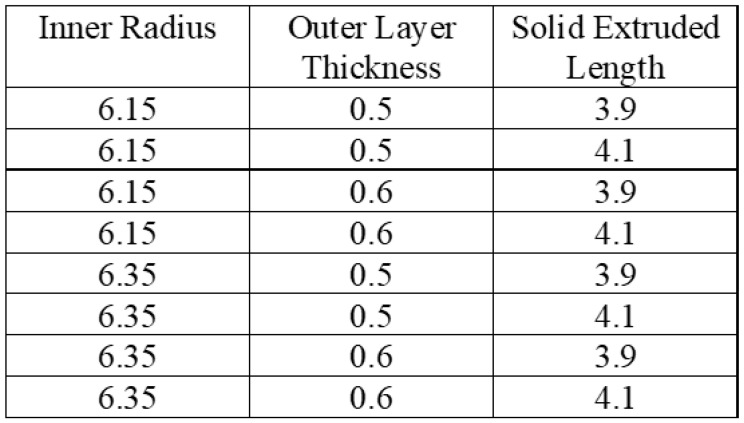
Full factorial design matrix with input parameter values.

Once more, ABAQUS/CAE is linked with Isight^®^ to conduct the DOE study, where Abaqus is added as the application component and DOE is added as the process component, as shown in Figure 13. The input parameters are selected as follows: inner radius of the tube, outer layer thickness of the tube and solid extruded length, which corresponds to the cavity length, as mentioned before. Output parameters are selected: maximum displacement of the tube and maximum stress of the tube. A full factorial technique is applied. A full factorial indicates experimental designs that contain all possible combinations of all levels of all factors, as shown in Figure 14. No combinations are excluded. A two-level, three-factor method is selected. The two levels chosen are the upper and lower values over the optimal values computed before. For inner radius: 6.15 and 6.35 mm. For outer layer thickness: 0.5 and 0.6 mm. For solid extruded length: 3.9 and 4.1, corresponding to cavity length of 86.1 and 85.9 mm, as shown in Figure 15. The full factorial method tries all the possible combinations of the given numbers and studies the individual and interactive effects of the parameters based upon the output parameters. The design of experiment results are presented in Section 3.3.

**Figure 16:**
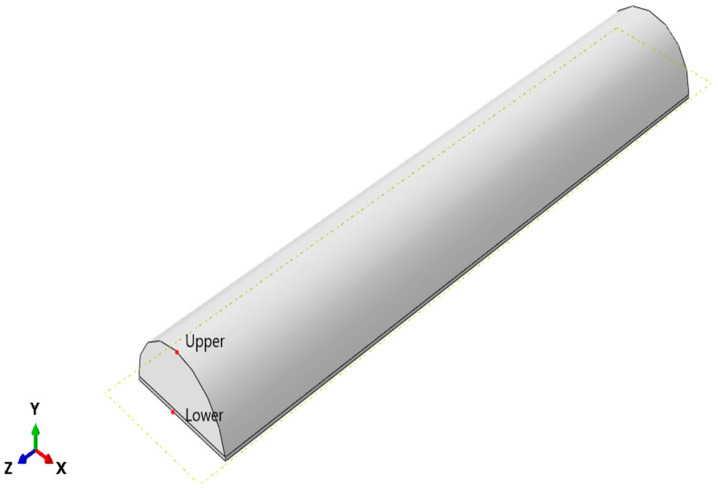
Points where sensors are defined to extract the displacement.

The FE model is now to be controlled through a PID control code written in FORTRAN language in a user subroutine called UAMP. First, ABAQUS/CAE is linked to Visual Studio (2019, Microsoft) and a Fortran complier so that the control code can be processed. A user subroutine UAMP is written, which takes sensors’ readings, computes the controller output, and then passes the new amplitude value. The sensors are set to take the displacement values of the two points at the face of the actuator, so as to measure the bending angle via Equation (1), as shown in Figure 16.

**Figure 17:**
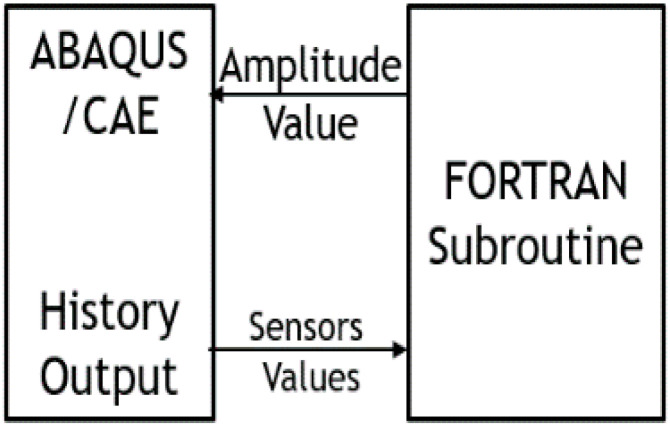
Workflow of the program.

First, ABAQUS/CAE is opened. Then, Fortran Compiler and Visual studio are invoked. The job is then submitted. The sensors’ values are passed to UAMP Subroutine (written in visual studio), which is compiled by the Fortran compiler. Subroutine returns amplitude value to ABAQUS. ABAQUS simulates and calculates the values at this time increment, and the process is repeated until simulation finishes, as shown in Figure 17.

**Figure 18:**
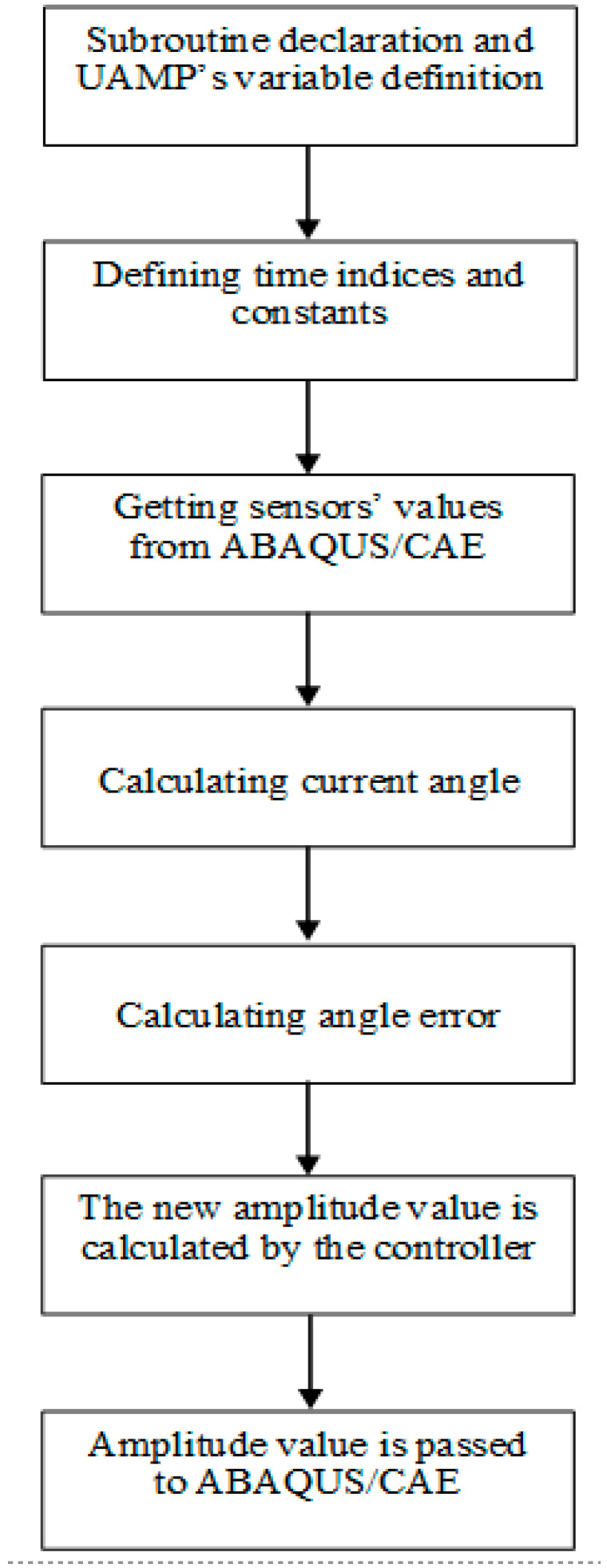
Flow chart of the UAMP subroutine.

The UAMP subroutine is written and divided as follows. The first part is subroutine declaration and UAMP’s variable definition. This part declares the UAMP subroutine and defines the variables that are passed to and from the subroutine. The second part is defining the time indices and the constants used in the code. The third part is getting the sensors’ values from ABAQUS/CAE and calculating the current angle. Then, the angle error is calculated and passed to the controller. The controller action is calculated, and a condition is set to limit the maximum amplitude given by the controller action, so that it does not exceed the specifications of the controller. Finally, the amplitude value is passed back to ABAQUS/CAE. This process is shown in Figure 18.

The sensors’ values are passed to the subroutine from ABAQUS. Z_Upper represents the z-displacement of the upper node. Z_Lower represents the z-displacement of the lower node. Y_Upper represents the y-displacement of the upper node. Y_Lower represents the y-displacement of the lower node. The current bending angle is calculated by the following equation:

CurrentAngle = 90 − ATAN((Z_Upper − Z_lower)/(Y_Lower − Y_Upper)) * 180/π(1)

**Figure 19:**
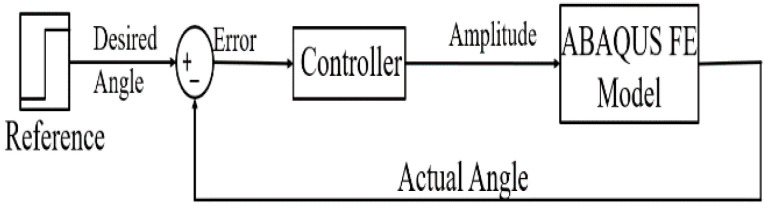
Closed loop block diagram.

Error in angle is calculated and controller action is determined to process the new amplitude value, which is passed to ABAQUS to proceed with the simulation. This loop is repeated each time step. The closed loop block diagram is shown in Figure 19. The full code used can be found in Appendix A.

## 3. Results

The results of all the processes performed on the model are presented in this section. In Section 3.1, the open loop response results are presented. In Section 3.2, optimization results are presented. Then, the design of experiment results are presented in Section 3.3. Finally, control-in-the-loop results are presented in Section 3.4

### 3.1. Open-Loop Simulation Results

**Figure 20:**
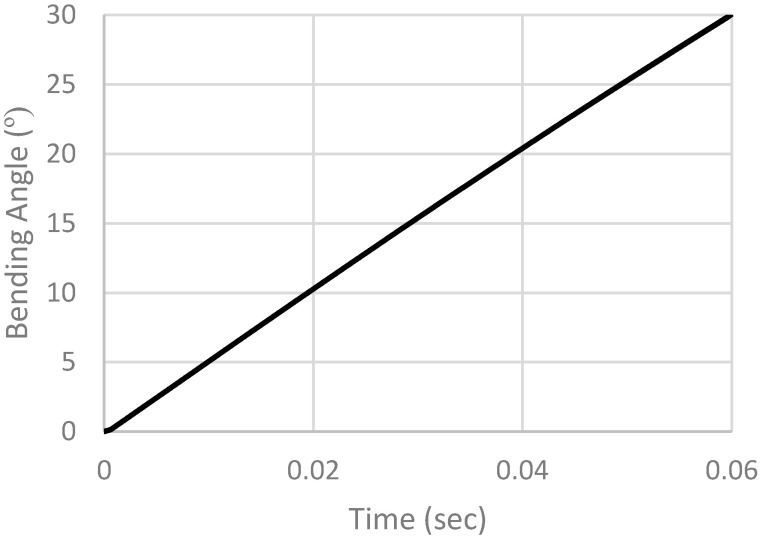
Open loop simulation results.

We focus on the bending angle of the actuator to evaluate its performance based on the achieved bending. The x–y data of any point can be extracted, and the bending angle is calculated from Equation (1). Figure 20 shows the open loop response of the actuator.

### 3.2. Optimization Results

#### 3.2.1. Dimensional Optimization Results

For the first model of dimensional optimization, with inner radius and solid extruded length as input parameters, the model is iterated 201 times for 33 h using a computer with 8GB RAM.

The optimum solution is of an inner radius of 6.25 mm and a solid extruded length of 4 mm, corresponding to a cavity length of 86 mm. The optimum solution corresponds to a maximum stress of 470.33 MPa and a maximum displacement of 100.58 mm.

Table 1 shows the linear correlation between input and output parameters. The solid extruded length has a small positive correlation with maximum stress and maximum displacement. The inner radius has high positive correlation values with both maximum stress and maximum displacement, as shown in Table 2.

For the second model of dimensional optimization, with inner radius and outer layer thickness as input parameters, the model is iterated 63 times for 47 h using a computer with 8 GB RAM**.**

**Figure 21:**
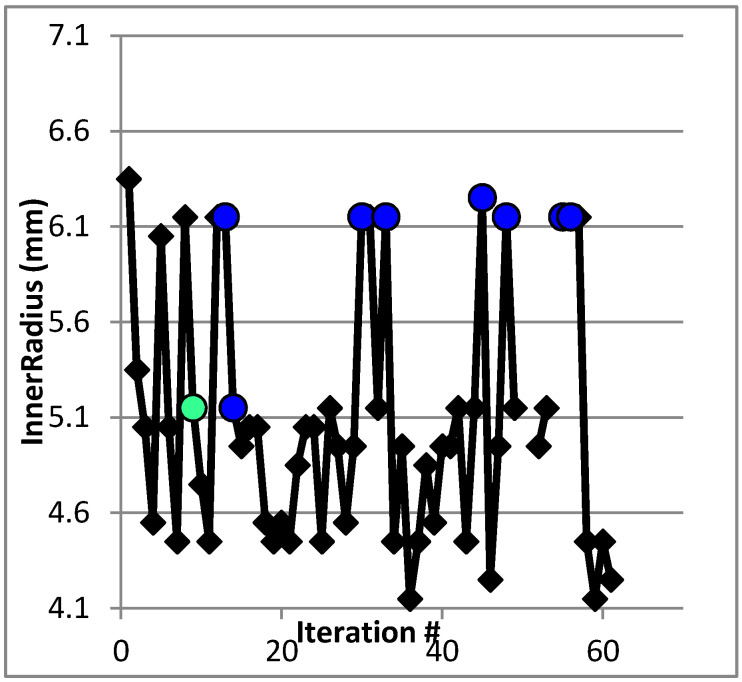
Inner radius values vs. iteration number for the second model.

It is noted that the computation time has increased. This is because when the outer layer’s thickness is changed, the model becomes more complex. The optimum design values were found to be 5.15 mm for the inner radius and 0.5 mm for the outer layer thickness, corresponding to maximum displacement and maximum stress values of 66.679 mm and 433.53 MPa, respectively. It is noted that one of the best cases, which is represented by the colored blue dots shown in Figure 21, is point 45, which has an outer layer thickness of 0.5 mm and an inner radius of 6.25 mm, which has the same inner radius as was obtained from the first model.

Table 2 shows the linear correlation between input and output parameters. The inner radius has a high positive correlation with maximum stress and small positive correlation with maximum displacement. The outer layer thickness and the maximum stress has a correlation value of approximately zero indicating that no correlation exists. Additionally, the outer layer thickness and the maximum displacement have high negative correlation.

#### 3.2.2. Material Optimization Results

For the material optimization model with inner and outer layer materials as input parameters, the model is iterated 48 times for 29 h using a computer with 8GB RAM. The optimum design case was found to be dragon-skin 20 for the inner layer material and EcoFlex 50 for the outer layer material. This case corresponds to a maximum stress of 473.02 MPa and a maximum displacement of 61.332 mm. 

Table 3 shows the linear correlation between input and output parameters. The outer layer material has low negative correlation with maximum displacement and maximum stress. The inner layer material has high negative correlation with both maximum displacement and maximum stress.

### 3.3. Design of Experiment Results

**Figure 22:**
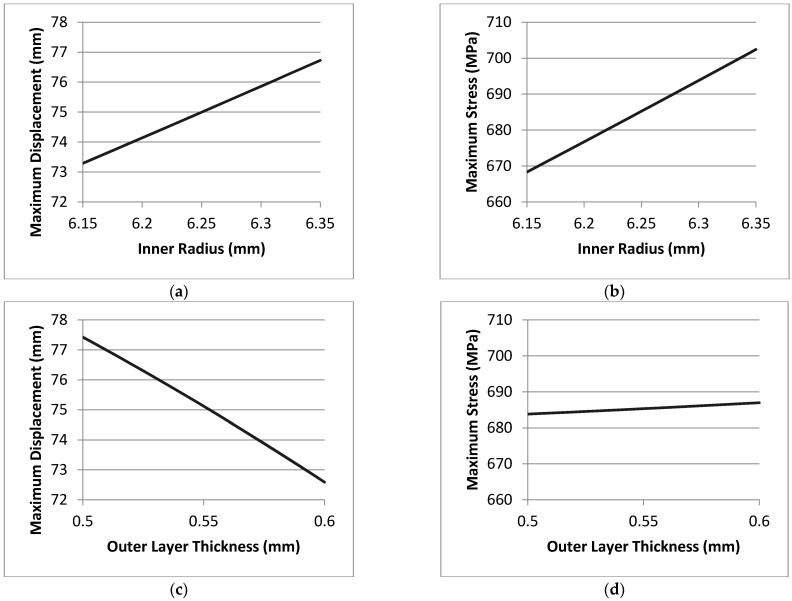

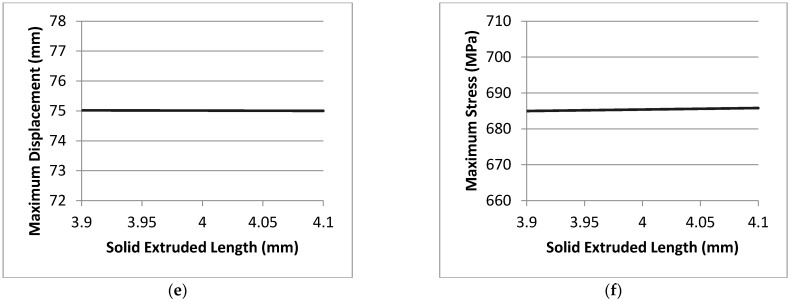
Design of experiment results and the changes in the output with respect to the input: (**a**) Maximum displacement vs. inner radius. (**b**) Maximum stress vs. inner radius. (**c**) Maximum displacement vs. outer layer thickness. (**d**) Maximum stress vs. outer layer thickness. (**e**) Maximum displacement vs. solid extruded length. (**f**) Maximum stress vs. solid extruded length.

The model is run, and results are extracted. The relative effects of the input parameters on the output responses are as shown in Table 4. The changes in the output with respect to the input are shown in Figure 22.

From the above results, it is noted that the inner radius has a significant effect on both maximum displacement and maximum stress. Cavity length is not a significant factor for both maximum displacement and stress. Additionally, outer layer thickness is not a significant factor for maximum stress. However, it has a negative effect on the maximum displacement. It is also noted that the interactive effects are not significant. The above plots demonstrate that if the solid extruded length is slightly changed, that would not affect the system. Additionally, if only the inner radius is slightly changed, that would also change the displacement and the stress in a directly proportional manner. If the outer layer thickness is slightly changed, it would not affect the stress but would only affect the displacement in an inversely proportional manner. However, the changes in the output values in response to the input values are minimal and within an acceptable range. Thus, the design is said to be robust.

### 3.4. Software-In-The-Loop Results

**Figure 23:**
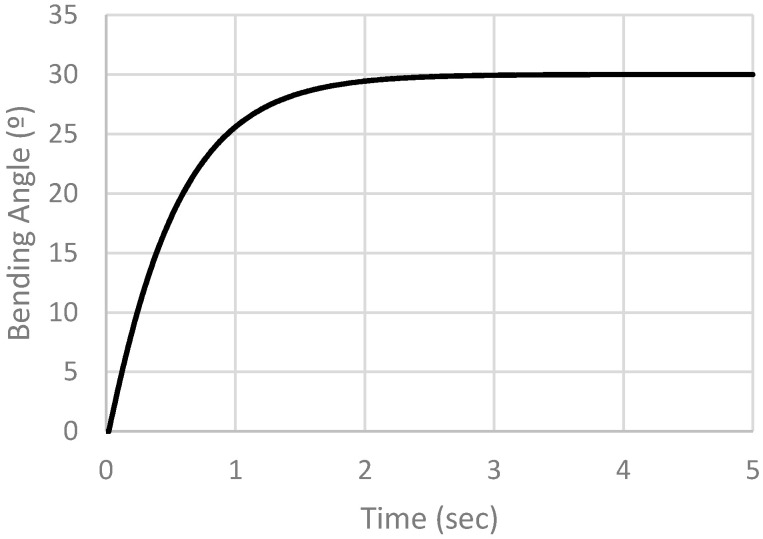
Simulation results of PID controller with Kp = 0.5, Ki = 0.05 and Kd = 0.001.

First, the controller’s parameters were selected to be Kp = 0.5, Ki = 0.05 and Kd = 0.001. The desired angle was set to be 30 degrees. The job was submitted, and the results were extracted. Bending angle in degrees is plotted against time in seconds, as shown in Figure 23. From the actuator response it is noticed that the rise time (tr) = 1.11 s, the settling time (ts) (5%) = 1.52 s and ts (2%) = 1.96 s. Root mean square (RMS) error is calculated by comparing the bending angle at each increment with the set value, and then calculating the RMS error by Equation (2), where current angle is the measured bending angle at time increments of 0.01 s. Set point is the desired angle of 30 degrees, and n is the number of readings. The RMS error is found to be equal to 7.0958397 degrees, corresponding to 23.6528%.

(2) $$\mathrm{RMS}\;\mathrm{Error}=\;\sqrt{\overset{n}{\underset{i=1}{\sum }}\frac{{\left(\mathrm{Cuurrent}\;\mathrm{Angle}-\mathrm{Set}\;\mathrm{Point}\right)}^{2}}{n}}$$

**Figure 24:**
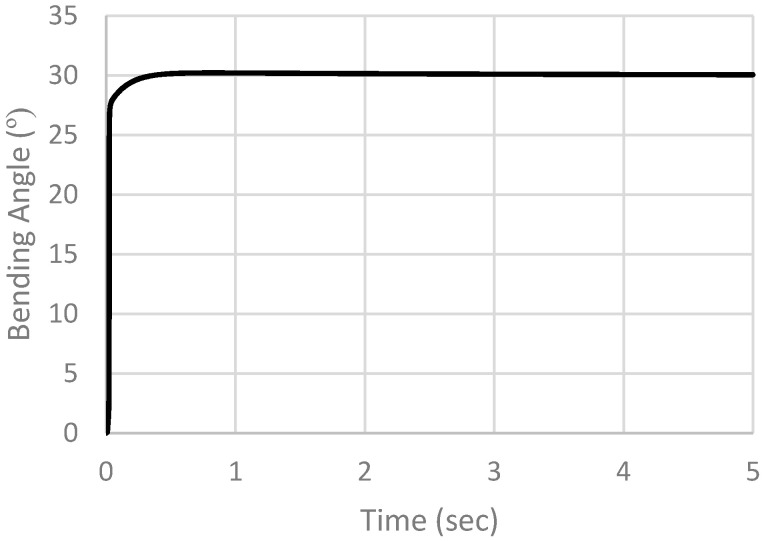
Simulation results of PI controller with Kp = 1.5, Ki = 0.5.

This response is noticeably slow. So, the controller’s parameters were tuned, and the new controller’s parameters were selected to be Kp = 1.5 and Ki = 0.5. The derivative action was removed, so we expect to see an overshoot. The job was resubmitted, and results were extracted, to be displayed in Figure 24. From the actuator response we notice that the rise time is now equal to 0.00647 s, the settling time (ts) (5%) = 0.094318 s and ts (2%) = 0.194318 s, with an overshoot of 0.20973 degrees, as expected. The root mean square (RMS) error is calculated and is equal to 0.322116338 degrees, corresponding to 1.07372%. Such a performance is better than the previous one in terms of characteristics and RMS error. So, PI controller is selected.

**Figure 25:**
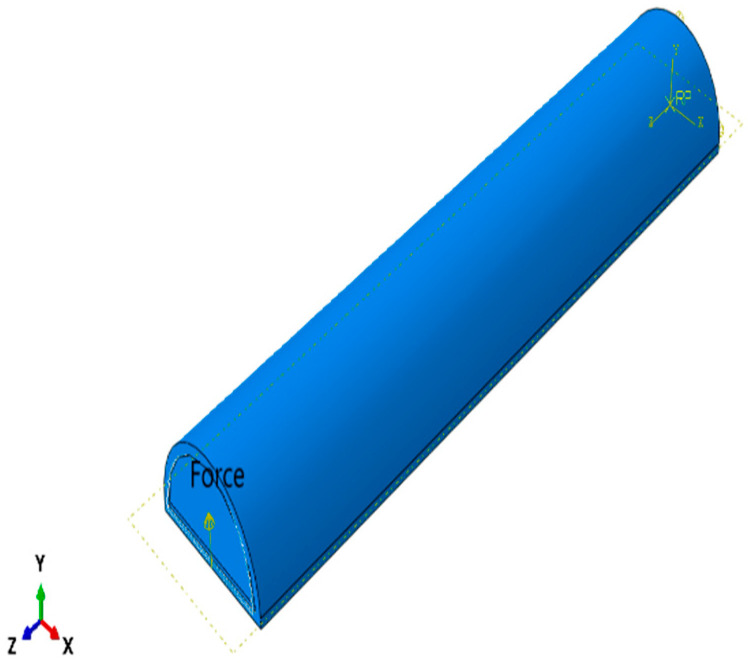
Concentrated force at the face of the SPA.

**Figure 26:**
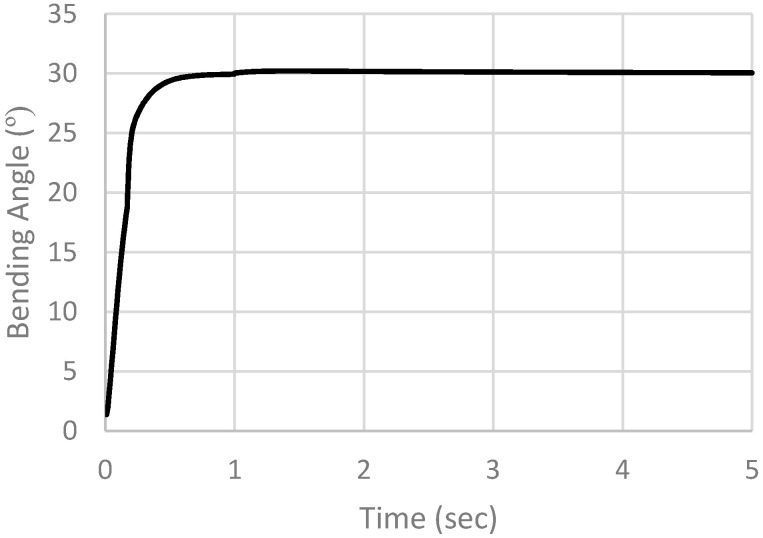
Simulation results with concentrated force added at the tip of the actuator.

After the first simulation, a concentrated force is added at the tip of the actuator to simulate the finger’s resistance to the actuator, as shown in Figure 25. The job is resubmitted, and results are extracted. Figure 26 shows the results of simulation. From the simulation results we notice that tr = 0.24594 s, ts (5%) = 0.3684 s and ts (2%) = 0.502 s.

## 4. Discussion

The results show that a soft pneumatic actuator with the optimized parameters can be implemented and controlled to achieve higher bending angles and apply higher forces. Using simulation tools enables us to analyze the performance of the actuator and study the results of our experiments to determine whether the design is robust or not. A soft pneumatic actuator with an inner radius of 6.25 mm, sa olid extruded length of 4mm and an outer layer thickness of 0.5 mm can achieve the highest bending and force needed. These parameters are the optimal parameters extracted from our simulation based on our objective function, which is to achieve maximum bending at the corresponding stress. The model is controlled by a code to test its response and reach the best controller parameters. After tuning, it is found that a PI controller with Kp = 1.5 and Ki = 0.5 has a lower rise time of 0.00647 s, a settling time (ts) (5%) = 0.094318 s and ts (2%) = 0.194318 s. Future work must include the fabrication of the soft pneumatic actuator with the optimal dimensional and material parameters to verify the model’s data extracted, and compare these versus the experimental data.

## Figures and Tables

**Table 1:** Correlation table of first model.

Name	Maximum Stress	Maximum Displacement
Solid Extruded Length	0.114523212	0.115576479
Inner Radius	0.843821803	0.847783115

**Table 2:** Correlation table of first model.

Name	Maximum Stress	Maximum Displacement
Inner Radius	0.991336239	0.443522174
Outer Layer Thickness	−0.000547	−0.813641802

**Table 3:** Correlation table of first model.

Name	Maximum Stress	Maximum Displacement
Outer Layer Material	−0.176361891	−0.475591571
Inner Layer Material	−0.978232335	−0.897922165

**Table 4:** The relative effects of the input parameters on the output responses.

Sources	Max Displacement	Max Stress
Inner Radius	40.531	87.472
Inner Radius–Outer Layer Thickness	−2.039	−0.32753
Inner Radius–Solid Extruded Length	0.18253	0.82108
Outer Layer Thickness	−56.962	8.1345
Outer Layer Thickness–Solid Extruded Length	0.063	1.1223
Solid Extruded Length	−0.22316	2.1222

## Data Availability

Not applicable.

## Appendix A

In the controlling of the model, a subroutine derives the sensor data from ABAQUS/CAE software and computes the value of the next amplitude before passing it back to ABAQUS/CAE. The code is as follows:

SUBROUTINE UAMP(

* ampName, time, ampValueOld, dt, nProps, props, nSvars,

* svars, lFlagsInfo,

* nSensor, sensorValues, sensorNames, jSensorLookUpTable,

* AmpValueNew,

* lFlagsDefine,

* AmpDerivative, AmpSecDerivative, AmpIncIntegral,

* AmpDoubleIntegral)

C

INCLUDE ‘ABA_PARAM.INC’

!DEC$ OBJCOMMENT LIB:”libeng.lib”

interface

function ENGOPEN (command) bind(C,name=“ENGOPEN”)

integer(INT_PTR_KIND()) :: ENGOPEN

character, dimension(*), intent(in) :: command

end function ENGOPEN

function mxCreateDoubleMatrix (a1,b1,c1) bind(C,name=“mxCreateDoubleMatrix”)

integer*8 :: a1,b1,c1

integer*8 :: mxCreateDoubleMatrix

intent(in) :: a1,b1,c1

end function mxCreateDoubleMatrix

function mxCreateDoubleScalar (a2) bind(C,name=“mxCreateDoubleScalar”)

real*8 :: a2

integer*8 :: mxCreateDoubleScalar

intent(in) :: a2

end function mxCreateDoubleScalar

Subroutine mxDestroyArray (a3)

cDEC$ ATTRIBUTES DECORATE,ALIAS:”mxDestroyArray” :: mxDestroyArray

integer*8, dimension(*) :: a3

end Subroutine mxDestroyArray

function mxGetPr(a4) result(ptr) bind(C,name=‘mxGetPr’)

import

implicit none

integer*8, dimension(*), intent(in) :: a4

integer*8 :: ptr

end function mxGetPr

Subroutine mxCopyReal8ToPtr (a5,b5,c5)

cDEC$ ATTRIBUTES DECORATE,ALIAS:”mxCopyReal8ToPtr” ::mxCopyReal8ToPtr

real*8 a5(*)

integer*8 b5

integer*8 c5

end Subroutine mxCopyReal8ToPtr

function engPutVariable (a6,b6,c6) bind(C,name=“engPutVariable”)

integer*8, intent(in) :: a6

character, dimension(*), intent(in) :: b6

integer*8, dimension(*), intent(in) :: c6

end function engPutVariable

function engEvalString (a7,b7) bind(C,name=“engEvalString”)

integer(INT_PTR_KIND()) :: engEvalString

integer*8, intent(in) :: a7

character, dimension(*), intent(in) :: b7

end function engEvalString

function engGetVariable (a8,b8) bind(C,name=“engGetVariable”)

integer*8 :: engGetVariable

integer*8, intent(in) :: a8

character, dimension(*), intent(in) :: b8

end function engGetVariable

Subroutine mxCopyPtrToReal8 (a9,b9,c9)

cDEC$ ATTRIBUTES DECORATE,ALIAS:”mxCopyPtrToReal8” ::mxCopyPtrToReal8

integer*8 a9

real*8 b9

integer*8 c9

end Subroutine mxCopyPtrToReal8

function engClose (a10) bind(C,name=“engClose”)

integer(INT_PTR_KIND()) :: engClose

integer*8, intent(in) :: a10

end function engClose

end interface

C time indices

parameter (iStepTime = 1,

* iTotalTime = 2,

* nTime = 2)

C flags passed in for information

parameter (iInitialization = 1,

* iRegularInc = 2,

* iCuts = 3,

* ikStep = 4,

* nFlagsInfo = 4)

C optional flags to be defined

parameter (iComputeDeriv = 1,

* iComputeSecDeriv = 2,

* iComputeInteg = 3,

* iComputeDoubleInteg = 4,

* iStopAnalysis = 5,

* iConcludeStep = 6,

* nFlagsDefine = 6)

dimension time(nTime), lFlagsInfo(nFlagsInfo),

* lFlagsDefine(nFlagsDefine)

dimension jSensorLookUpTable(*)

dimension sensorValues(nSensor), svars(nSvars), props(nProps)

character*80 sensorNames(nSensor)

character*80 ampName

real(8) :: integral

real(8) :: derivative

real(8) :: Z

real(8) :: Y

real(8) :: TAN

parameter( kp=0.02d0, ki =0.01d0,

*  kd=0.01d0,

* pi=4 * atan (1.0_8), TARGET_ANGLE = 30.0d0)

! Check point 1:

print *, ‘time=‘, time(1)

Y_LOWER = GETSENSORVALUE(‘Y_LOWER’,

* jSensorLookUpTable,

* sensorValues)

C

Z_LOWER = GETSENSORVALUE(‘Z_LOWER’,

* jSensorLookUpTable,

* sensorValues)

C

Y_UPPER = GETSENSORVALUE(‘Y_UPPER’,

* jSensorLookUpTable,

* sensorValues)

C

Z_UPPER = GETSENSORVALUE(‘Z_UPPER’,

* jSensorLookUpTable,

* sensorValues)

C

C

tim=time(1)

IF(lFlagsInfo(iInitialization) .EQ. 1) THEN

  ampValueNew = 0

counter = 0

  lFlagsDefine(iConcludeStep) = 0

ELSE if(tim.EQ.0.01d0) then

ampValueNew=0.02

ANGLE_ERROR=30

counter = svars(1)

ELSE

counter = svars(1)

Z = (Z_UPPER - Z_LOWER)

Y = (Y_LOWER - Y_UPPER)

CURRENT_ANGLE= 90.0d0-(ATAN(Z/Y)*180/pi)

ANGLE_ERROR = (TARGET_ANGLE - CURRENT_ANGLE)/TARGET_ANGLE

print *, ‘dt=‘, dt

print *, ‘ANGLE_ERROR=‘, ANGLE_ERROR

print *, ‘Current_ANGLE=‘, CURRENT_ANGLE

integral = 0.05*(ANGLE_ERROR + svars(2))*0.01

derivative = 0.001*(ANGLE_ERROR - svars(3))*100

OUTPUT_VALUE=0.5*ANGLE_ERROR+integral

print *, ‘OUTPUT_VALUE=‘, OUTPUT_VALUE

svars(2) = integral

svars(3) = ANGLE_ERROR

if(OUTPUT_VALUE .GE. 1) then

ampValueNew = ampValueOld+0.04

ELSE IF(OUTPUT_VALUE .LE. 0) THEN

ampValueNew =AMPVALUEOLD

ELSE

ampValueNew =AMPVALUEOLD+OUTPUT_VALUE*0.04

ENDIF

  IF(ANGLE_ERROR .EQ. 0) THEN

    lFlagsDefine(iConcludeStep) = 1

  END IF

END IF

svars(1) = counter + 1

svars(2) = integral

svars(3) = ANGLE_ERROR

print *, ‘amp=‘, AMPVALUENEW

print *, ‘integral=‘, integral

print *, ‘derivative=‘, derivative

print *, ‘counter=‘, counter

C

return

end
